# Green Tea Catechins: Their Use in Treating and Preventing Infectious Diseases

**DOI:** 10.1155/2018/9105261

**Published:** 2018-07-17

**Authors:** Wanda C. Reygaert

**Affiliations:** Biomedical Sciences Department, Oakland University William Beaumont School of Medicine, Rochester, MI 48309, USA

## Abstract

Green tea is one of the most popular drinks consumed worldwide. Produced mainly in Asian countries from the leaves of the* Camellia sinensis *plant, the potential health benefits have been widely studied. Recently, researchers have studied the ability of green tea to eradicate infectious agents and the ability to actually prevent infections. The important components in green tea that show antimicrobial properties are the catechins. The four main catechins that occur in green tea are (-)-epicatechin (EC), (-)-epicatechin-3-gallate (ECG), (-)-epigallocatechin (EGC), and (-)-epigallocatechin-3-gallate (EGCG). Of these catechins, EGCG and EGC are found in the highest amounts in green tea and have been the subject of most of the studies. These catechins have been shown to demonstrate a variety of antimicrobial properties, both to organisms affected and in mechanisms used. Consumption of green tea has been shown to distribute these compounds and/or their metabolites throughout the body, which allows for not only the possibility of treatment of infections but also the prevention of infections.

## 1. Introduction

Infectious diseases are a leading cause of morbidity and mortality worldwide. HIV/AIDS and malaria are among the top ten infectious diseases in the world; and the most common types of infections are respiratory tract and diarrheal diseases [1]. With the advent of antimicrobial agents in the mid-1900s came the hope that eradication of infectious diseases was close. Unfortunately, the microorganisms involved were able to become resistant to the antimicrobial agents, and that only made it harder to fight these organisms. The CDC has estimated that each year more than two million people in the US suffer from antibiotic-resistant infections and that as many as 23,000 people die each year from these infections [2]. This results in not only increased morbidity and mortality, but also increased healthcare costs, which can be a huge financial burden for many countries. A recent analysis of the medical costs from healthcare-associated infections (those infections acquired in a healthcare facility) alone estimated that the annual costs of these infections in the US are between 28 and 45 billion dollars [3]. Antimicrobial resistance issues continue to impact these costs. One study found that the cost of antimicrobial resistance associated illnesses in the US could be as high as 55 billion dollars (20 billion dollars for healthcare costs and 35 billion dollars for lost productivity) annually [4]. To help in the fight against infectious diseases, researchers are looking at the possibilities of using natural plant products, which could turn out to provide a tremendous cost savings in healthcare. One of the plants that is currently being widely studied is the tea plant, looking especially at green tea.

Tea is one of the most commonly consumed beverages in the world, and green tea is becoming increasingly popular, accounting for around 20% of total global tea production. Tea is produced from the* Camellia sinensis* plant and is grown in over 30 countries. The best areas for growing tea plants are in specific tropical and subtropical regions. There are four main tea types produced: white, green, Oolong, and black tea. The type of tea is determined by how the tea leaves are processed, specifically by drying and fermentation methods. White tea is processed the least and uses very young leaves and leaf buds. Green tea is produced from more mature leaves with no fermentation. Oolong tea is produced by partially fermenting the leaves and black tea by fully fermenting the leaves [5–7]. Green tea is most commonly consumed in China, Japan, and Korea. Black tea is most commonly consumed in the US and the UK [8].

Green tea has been shown to have anticarcinogenic, anti-inflammatory, antimicrobial, and antioxidant properties and is beneficial in cardiovascular disease (CVD), diabetes and obesity, and neurologic and oral health. The anticarcinogenic properties include controlling cell proliferation, apoptosis and angiogenesis in tumor cells [9–12]. Inflammation is a component of many conditions and diseases including aging, arthritis, cancer, CVD, diabetes, and obesity. The general anti-inflammatory properties of green tea include the ability to decrease the denaturation of proteins and increase the production of anti-inflammatory cytokines [7, 13]. Oxidative stress results from the damaging effects of reactive oxygen species (ROS). The antioxidant properties of green tea include the ability to limit the amount of free radicals by binding to ROS, upregulating basal levels of antioxidant enzymes, and increasing the activity of these antioxidant enzymes [6, 14, 15]. The effects of green tea on CVD include the anti-inflammatory and antioxidant effects. In addition, the consumption of green tea has been shown to inhibit atherosclerosis, reduce total lipid levels, and improve the ratio of LDL to HDL [16, 17]. Diabetes and obesity are closely associated with a spectrum of disorders known as metabolic syndrome (MetS) which includes increased waist diameter, elevated plasma triglycerides, decreased HDL, increased fasting blood glucose, and elevated blood pressure [18, 19]. Type 2 diabetes is also associated with insulin resistance and sometimes decreased insulin production. Green tea has been shown to increase insulin receptor sensitivity and stimulate glucose-induced insulin secretion [20, 21]. Obesity is a result of an increase in fat mass which is caused by increase in the size of fat cells. Green tea has been shown to inhibit digestive enzymes and absorption of fat, which leads to decreased body waist circumference, intra-abdominal fat, plasma total and LDL cholesterol, triglycerides, and blood pressure [22–24]. The challenges of inflammation and oxidative stress can lead to DNA damage, protein misfolding, and loss of ATP production in mitochondria. This can result in cell death and loss of cognitive functions in the brain. The anti-inflammatory and antioxidant properties of green tea also protect neurons, and green tea metabolites have been shown to cross the blood brain barrier [25–29]. Green tea has been shown to be antimicrobial against most oral bacteria. In addition, it has been shown to improve oral health by increasing the activity of oral peroxidases, preventing the development and progression of periodontitis, and reducing dentin erosion and tooth loss, and it has a role in improving bad breath [30–34].

## 2. Green Tea Composition

The components in green tea that are the most medically relevant are the polyphenols. The most pertinent polyphenols are the flavonoids; and the most pertinent flavonoids are the catechins. The catechins comprise 80-90% of the flavonoids and around 40% of the water-soluble solids in green tea. Green tea contains more catechins than the other teas, mainly because of the way it is processed after harvesting. The amount of catechins in green tea can also be affected by where the tea is grown, the growth conditions, when it is harvested, how the leaves are processed, and the brewing temperature and length of time of brewing. These factors lead to a huge variation in catechin content among the varieties and brands of green tea consumed [35–45].

The four main catechins found in green tea are (-)-epicatechin (EC), (-)-epigallocatechin (EGC), (-)-epicatechin-3-gallate (ECG), and (-)-epigallocatechin-3-gallate (EGCG). The most abundant catechin is EGCG (~60%), and the next most abundant is EGC (~20%), then ECG (~14%), and EC (~6%). EGCG is the most studied in association with health, but EGC and ECG have been studied as well. As mentioned above, there can be a wide variation in the amount of catechins in any particular green tea beverage, although standardized extracts are available for use as supplements [7, 46, 47].

In order to be effective in the body these catechins need to be bioavailable after consumption. Once in the body, the catechins undergo metabolic processing in the liver and small intestine and colon. This processing produces glucuronide and sulfate conjugates or methyl epicatechins. Native forms of ECG and EGCG and metabolites of EC and EGC can be detected and measured in blood plasma. No forms of ECG and EGCG can be detected in urine, only metabolites of EC and EGC [48, 49]. Catechins are generally most stable in solution at a pH range of 4-6. It is now known that human serum albumin acts as a stabilizer, binding to the catechins and then transporting them [50]. Various studies in humans have found that the peak concentrations of catechins and their metabolites occur in blood plasma between 1.5 and 2 hours after ingestion and in urine between 4 and 6 hours after ingestion. The levels of these peak concentrations are affected by an individual's metabolism and of course by the amount of catechins in the ingested type of green tea. Commonly, the levels found in the body are directly proportional to the amount of catechins consumed [51–53]. Tables 1 and 2 show examples of blood plasma and urine concentration studies in humans.

## 3. Antimicrobial Properties

The antimicrobial effects of green tea catechins (GTCs) on microorganisms have been studied for many years. Green tea has been shown to combat these organisms in various ways, directly and indirectly, and has been shown to work synergistically with some antibiotic agents. Other known health benefits of green tea such as the anti-inflammatory and antioxidant effects may also contribute to the antimicrobial effects. Studies conducted on* Escherichia coli* found that exposure to green tea polyphenols (GTPs) resulted in major gene expression changes for 17 genes, with upregulation occurring in nine genes and downregulation in eight genes [75–77].  Table 3 shows a summary of the antimicrobial effects of green tea on bacteria.

## 4. Effects on the Bacteria Cell Membrane

One of the major properties of GTCs is the ability to bind to bacterial cell membranes. This binding can lead to interference in various bacterial processes and can damage the cell membrane resulting in increased permeability and leading to cell lysis. Because EGCG is negatively charged it can combine with the positively charged bacterial cell membrane, especially in gram positive bacteria. The lipopolysaccharide (LPS) on the outer membrane of gram negative bacteria makes them more resistant to binding by GTCs [53, 63, 64, 66]. Studies with* E. coli* and* Pseudomonas aeruginosa* have shown that EGCG binding to the bacterial cell membrane can result in generation of H_2_O_2_ which is involved in damage to the cell membrane [63, 74]. Studies with* Staphylococcus aureus* have shown that this assault on the cell membrane causes a major cell wall stress response, resulting in upregulation of peptidoglycan biosynthesis genes and an alteration in cell wall structure. In methicillin-resistant* Staphylococcus aureus* (MRSA) strains, this change in peptidoglycan biosynthesis genes results in the production of PBP2 (penicillin-binding protein 2), which is what confers resistance to *β*-lactam drugs. Production of PBP2 is also inhibited by EGCG [64, 78, 79]. An important result of green tea binding is the loss of bacterial ability to bind to host cells. Studies using human and mammalian cells lines have shown that various bacteria such as* Fusobacterium nucleatum*,* Staphylococcus epidermidis*, and* Helicobacter pylori* have significantly decreased adherence to these cells [66, 67, 80]. Other important results are the loss of the ability for quorum sensing and biofilm formation of* P. aeruginosa*,* F. nucleatum*, and* Streptococcus mutans* [66, 68, 81]. Damage to the cell membrane also results in loss of function to transmembrane transporter proteins which are responsible for secretion of toxins and efflux of substances such as antimicrobial agents [53, 65, 69, 70].

## 5. Effects on Other Bacterial Cell Functions

There are a wide variety of other effects that GTCs have on bacterial functions. An important one which can affect most bacteria is the ability of GTCs to inhibit bacterial fatty acid biosynthesis by inhibiting enzymes involved in the biosynthetic pathway. Because this is an essential pathway for most bacteria, researchers are looking at targeting this pathway in antimicrobial drug development. Fatty acids are important for building cell membranes, as an energy source, and are involved in the production of toxic bacterial metabolites [53, 73]. Another target is the folate biosynthesis pathway. The enzyme dihydrofolate reductase (DHFR) is essential in this pathway, and is known to be a target for certain sulfa drugs. EGCG has also been shown to inhibit DHFR activity [53, 82]. Other important effects against enzymes include inhibition of bacterial DNA gyrase, inhibition of bacterial ATP synthase activity, and inhibition of bacterial protein tyrosine phosphatase and cysteine proteases [53, 71, 83]. Some specific bacterial effects include reducing bacterial H_2_S production and inhibiting hemolytic activity of* F. nucleatum*, inhibiting the ability of* Listeria monocytogenes* to escape from the macrophage phagosome by inhibiting activity of listeriolysin O, and inhibiting the ability of* E. coli* to transfer plasmid content via conjugation [66, 72, 84].

## 6. Synergism

Since GTCs are known to have antimicrobial action, researchers have begun assessing the potential synergism of these catechins with other known antimicrobial agents. Green tea catechins have now been shown to act in synergy with imipenem against MRSA; with metronidazole against* Porphyromonas gingivalis*; with azithromycin, cefepime, ciprofloxacin, chloramphenicol, doxycycline, erythromycin, nalidixic acid, piperacillin, or tobramycin against* E. coli*; with ampicillin, Cefalotin, doxycycline, erythromycin, penicillin, or tetracycline against* Enterobacter aerogenes*; with chloramphenicol or tetracycline against* Pseudomonas aeruginosa*; and with aztreonam, ceftazidime, ciprofloxacin, gentamicin, meropenem, or tetracycline against* Acinetobacter baumannii*. The ability of GTCs to inhibit the function of bacterial efflux pumps (as mentioned previously) also plays a role in at least an additive antimicrobial effect for GTCs and many antimicrobial drugs, especially in gram negative bacteria that possess RND-type efflux pumps [53, 69, 70, 85–89].  Table 4 lists antimicrobial agents that have shown synergy with GTCs and the targets of these drugs.

## 7. Effects on Other Microorganisms

Green tea catechins have also been shown to be effective against a number of viruses, parasites, fungi, and even prions. The main antiviral effects include inhibiting the virus from binding to and entering host cells (adenovirus, enterovirus, HBV, HCV, HIV, HSV, influenza, and rotavirus); inhibiting viral RNA and DNA synthesis and viral gene transcription (enterovirus, EBV, HBV, HCV, and HIV); and destroying and functionally altering various viral molecules (adenovirus, HSV, and influenza) [64, 90–96]. Studies performed with adult healthcare workers to determine if green tea supplements could prevent infection with viruses causing influenza showed significantly fewer instances of influenza symptoms and a reduced incidence of laboratory-confirmed influenza cases versus the control group [97].

The main effect of GTCs on various parasite infections is a decrease in parasite numbers and growth. Other effects noted were fragmentation of parasite DNA and reduced fatty acid synthesis in the parasites. Studies with parasites include* Plasmodium falciparum*,* Babesia *spp.,* Trypanosoma brucei*,* Trypanosoma cruzi*, and* Leishmania braziliensis* [98–102].

Fungi that have been affected by GTCs include* Aspergillus niger*,* Candida *spp.,* Penicillium *sp.,* Microsporum canis*,* Trichophyton mentagrophytes*, and* Trichophyton rubrum*. Research testing for synergistic effects found that EGCG showed synergism with amphotericin B, fluconazole, and miconazole in* Candida *spp.; and in* Candida tropicalis* strains that were resistant to fluconazole, EGCG, and fluconazole together induced apoptosis in the yeast cells [64, 103–107].

Prions are proteins that are considered to be infective agents because the abnormally structured (*β*-sheet) forms are able to induce normally structured (*α*-helix) forms to change shape. In the abnormal shape, protein function is lost and protein aggregation occurs in cells. Unlike other infectious agents, prions cannot be destroyed using autoclaving; the proteins have to be degraded to be noninfectious. Research using yeast cells found that EGCG could inhibit the *β*-sheet prions from changing the *α*-helical forms and could induce reversal of the *β*-sheet forms back to *α*-helical forms [108].

## 8. Antimicrobial Scope

There is a large amount of research that has assessed the antimicrobial effects of green tea catechins on a wide variety of microorganisms, including many gram negative and gram positive bacteria, some viruses, fungi, and prions. One of the most clinically important bacteria that has been researched is* S. aureus*, especially MRSA strains. The most studied gram negative bacteria is* E. coli* which is known for causing the majority of urinary tract infections. There are several recently published manuscripts that contain extensive information on which organisms are affected by green tea catechins [53, 64, 96, 109].

## 9. Prevention of Infection

Since it has been shown that GTCs have multiple types of antimicrobial abilities against so many organisms, it would be expected that green tea catechins could also prevent infections. One study was mentioned previously describing how green tea reduced the number of colds and influenza incidents. Another study involving adults showed that consuming green tea supplements twice daily for 3 months resulted in 32% fewer instances of cold or influenza symptoms and nearly 23% fewer illnesses of 2 or more days duration [110]. A study involving children found that, in school-aged children who consumed green tea on a regular basis, the number of incidents of influenza A or B was inversely associated with the number of cups of green tea consumed per day or per week [111]. Another study with Japanese nursey school children who gargled with green tea (or placebos) at least once each day found that there were up to 3 times fewer instances of illnesses with fevers in the green tea gargling group [112]. Two other studies with adults found that gargling with a green tea extract (GTE) solution resulted in at least half as many cases of influenza in the GTE gargling groups compared with the control groups [113, 114].

## 10. Conclusions

The research into the effects of green tea on human health has shown that it can be an important dietary factor in the prevention and treatment of various diseases such as arthritis, cancer, CVD, diabetes and obesity, infections, and in neurologic and oral health. Studies that were originally performed in animals and cell lines have become more frequently performed using humans. This type of research is vital if we are to fully discover what benefits GTCs can have in health issues. The more researchers that become involved in this, the clearer the answers. The studies on antimicrobial effects are providing very promising data, especially if GTCs prove to have synergistic abilities with many of the currently used antimicrobial agents and perhaps with drugs used to treat other diseases. The emergence of various multidrug-resistant bacteria, along with a dearth of effective antimicrobial drugs, makes the potential of green tea an extremely timely issue. There are also many areas across the globe where the cost of drugs is currently beyond the earning power of most of the population. Green tea is relatively inexpensive and fairly easy to obtain for most people. It could prove to be an answer for improving health on a global scale.

## Figures and Tables

**Table 1:** Amount of EGCG in blood plasma after a single dose.

EGCG dose (mg)	C_max_ (ng/ml)	Reference
200	73.7	[54]
400	111.8	
600	169.1	
800	438.5	

400	137.6	[55]
800	234.9	

88	135	[56]
140	34.7	[57]

225	300	[58]
375	1970	
525	2020	

50	130.4	[59]
100	180.4	
200	332.2	
400	624.5	
800	1067.4	
1600	3391.6	

200	376.9	[60]
400	525.2	
800	1682.1	

110	119	[61]
219	326	
329	321	

**Table 2:** Amount of EGC in 24-hour urine collection.

EGC dose (mg)	Amount in 24 hour urine (mg)	Reference
37	0.4	[54]
74	1.4	
111	3.5	
148	3.7	

82	~3.0	[56]
154	3.5	[57]
148	10.5	[62]

102	~3.0	[61]
204	~4.0	
306	~4.8	

**Table 3:** Antimicrobial effects of green tea catechins.

Organism	Effects	References
Cell Membrane	Binding to bacterial cell membrane	[63–65]
Associated Effects	Damaging bacterial cell membrane	[63]
	Inhibits ability of bacteria to bind to host cells	[66, 67]
	Inhibits ability of bacteria to form biofilms	[66, 68, 69]
	Disrupts bacterial quorum sensing	[68]
	Interferes with bacterial membrane transporters	[65, 69, 70]

Bacterial Cell Functions	Inhibits bacterial DNA gyrase	[71]
Effects	Reduces bacterial H_2_S production	[66]
	Inhibits bacterial hemolytic action	[66, 72]
	Inhibition of bacterial DHFR enzyme	[53]
	Inhibits bacterial fatty acid synthesis enzymes	[73]
	Increases bacterial internal ROS levels	[74]

**Table 4:** Synergism of green tea with antimicrobial agents.

Antimicrobial Action	Drug Synergism
Inhibit Cell Wall Synthesis	ampicillin
	ampicillin/sulbactam
	amoxicillin
	aztreonam
	cefalotin
	cefepime
	cefotaxime
	ceftazidime
	imipenem
	meropenem
	oxacillin
	penicillin
	piperacillin

Inhibit Protein Synthesis	amikacin
	azithromycin
	chloramphenicol
	doxycycline
	erythromycin
	gentamicin
	tetracycline
	tobramycin

Inhibit Nucleic Acid Synthesis	ciprofloxacin
	levofloxacin
	metronidazole
	nalidixic acid
